# Intestinal fibrosis is associated with lack of response to Infliximab therapy in Crohn's disease

**DOI:** 10.1371/journal.pone.0190999

**Published:** 2018-01-24

**Authors:** Jessica R. de Bruyn, Marte A. Becker, Jessica Steenkamer, Manon E. Wildenberg, Sybren L. Meijer, Christianne J. Buskens, Willem A. Bemelman, Mark Löwenberg, Cyriel Y. Ponsioen, Gijs R. van den Brink, Geert R. D’Haens

**Affiliations:** 1 Department of Gastroenterology and Hepatology, Academic Medical Center, Amsterdam, The Netherlands; 2 Tytgat Institute for Liver and Intestinal Research, Academic Medical Center, Amsterdam, The Netherlands; 3 Department of Pathology, Academic Medical Center, Amsterdam, The Netherlands; 4 Department of Surgery, Academic Medical Center, Amsterdam, The Netherlands; University Hospital Llandough, UNITED KINGDOM

## Abstract

**Introduction:**

Overt fibrostenotic disease is a relative contraindication for anti-TNF therapy in Crohn’s disease. We hypothesized that subclinical fibrosis may also contribute to an incomplete response to anti-TNF therapy before the onset of symptomatic stenosis.

**Methods:**

In a previous trial, patients with ileocecal Crohn’s disease were randomized to either immediate ileocecal resection or medical treatment with Infliximab. In case of insufficient response to Infliximab, the latter underwent secondary ileocecal resection. We compared specimens from those patients undergoing immediate resection (Infliximab naïve, n = 20) to those who failed Infliximab therapy (n = 20).

**Results:**

Infliximab naïve and Infliximab failure patients had similar severity of inflammation when assessed by CRP levels (median 14 vs 9 mg/L) and histology (Geboes-D’Haens-score, median 10 vs 11 points). On immunohistochemistry, collagen-III and fibronectin depositions were increased in patients previously exposed to Infliximab compared to patients naïve to Infliximab. On mRNA level, procollagen peptidase showed significantly more mucosal mRNA expression in Crohn’s disease patients who failed Infliximab. Infliximab responders showed no increase of this marker after 4 weeks of successful Infliximab treatment.

**Discussion:**

Failure to Infliximab therapy is associated with subclinical fibrosis in Crohn’s disease.

## Introduction

Crohn’s disease is characterized by chronic intestinal inflammation and tissue injury leading to dysregulation of wound healing, unrestrained proliferation of mesenchymal cells and an excessive accumulation of extracellular matrix (ECM) components such as collagen and fibronectin [1–3]. This aberrant wound healing process in combination with progressive contraction of the ECM eventually leads to fibrosis at the site of inflammation in the majority of patients [1, 2]. Obstructive symptoms caused by strictures is the clinical end stage of fibrosis and is observed in more than 40% of patients with Crohn’s disease [4]. The vast majority of these patients will need at least one surgical resection [5].

Monoclonal antibodies against tumor necrosis factor alpha (TNFα) have significantly improved the therapeutic options for Crohn’s disease patients. Infliximab (IFX) is an anti-TNFα antibody that blocks soluble and membrane bound TNF, induces lamina propria T-cell apoptosis and M2 type wound-healing macrophages [6–8]. Due to these properties, IFX suppresses the inflammatory response and contributes to rapid healing of the damaged intestinal tissue. Currently, IFX is the most effective therapy to induce and maintain mucosal healing in CD, with sustained complete mucosal healing rates of approximately 30% when used in monotherapy [9, 10].

Due to the anti-inflammatory actions of anti-TNF therapy, purely inflammatory strictures may improve with it. However, almost all strictures in Crohn’s disease also contain a fibrotic component, often complemented with prestenotic dilatation [11, 12]. Therefore fibrotic, stricturing disease is often considered as a relative contraindication for anti-TNF therapy [13], although data from a recent study suggest that Adalimumab may be effective in stricturing disease [14]. In fact, a small study where Crohn’s disease patients with stenotic disease were treated with IFX to evaluate the effect of anti-TNF on strictures, had to be terminated prematurely because of high need for surgery [15]. In line with a negative correlation between response to anti-TNF therapy and stenotic disease, the requirement for surgical interventions for patients with intestinal strictures has not changed in the past 25 years [16].

Due to the poor outcome of anti-TNF therapy in fulminant stricturing disease, we hypothesized that even subclinical fibrosis (i.e. that has not yet led to stenotic obstructive disease) may contribute to incomplete response to IFX. We aimed to investigate this hypothesis in Crohn’s disease patients who had failed or were naive to IFX therapy.

## Material and methods

### Patient selection

Between 2007 and 2014, patients with active recurrent Crohn’s disease of the terminal ileum failing thiopurine treatment were randomized to additional medical therapy with IFX or ileocecal resection in the Academic Medical Center in Amsterdam, the Netherlands (LIRIC trial, NTR1150, [17]). Patients who had prestenotic dilatation with fibrostenosing disease on screening magnetic resonance enterography were excluded from the trial. Patients who were randomized to IFX treatment, but did not respond to this treatment based on reappearance or continuation of symptoms as demonstrated by endoscopy or radiology, underwent subsequent ileocecal resection as routine care treatment. For each patient, age at operation, gender, disease duration, Montreal classification, smoking status, time of previous thiopurines treatment, duration of IFX treatment (if applicable), and serum CRP and albumin levels were collected pre-operatively.

For this study, resection specimens were retrieved form the pathology tissue bank and processed as described below. The most inflamed area was identified by an expert IBD-pathologist who was blinded for treatment allocation (SM). This most inflamed area was located in the terminal ileum in all specimens. Moreover we collected ileal biopsies from a second cohort of 5 Crohn’s disease patients before IFX treatment and from the same patient after 4 weeks of treatment. Those patients responded to IFX based on absence of ulcerations on endoscopy.

The medical ethical committee granted a waiver for this study based on Dutch legislation.

### Histology and immunohistochemistry

Immediately following surgery, ileocaecal resection specimens were routinely processed by the department of pathology for histology. This included a protocolized approach following national guidelines with extensive sampling of the affected and non-affected tissue perpendicular to the bowel wall and embedded in paraffin [18]. 4 μm thick sections were prepared and stained routinely for haematoxylin and eosin (H&E). Consecutive slides were used for immunohistochemistry. For immunohistochemistry, sections were deparaffinized, dehydrated and immersed in 0.3% H_2_O_2_ in methanol. After antigen retrieval and blocking, slides were incubated with the primary antibody dissolved in phosphate-buffered saline with 0.1% Triton X-100 and 1% bovine serum. To detect the ECM component fibronectin an anti-fibronectin antibody (ab 23750; Abcam, Cambridge, UK; dilution 1:200) was used; and to detect specific collagen deposition an anti-collagen I and anti-collagen III antibody (respectively 1310–01 and 1330–01, both from Southern Biotech, Birmingham, AL, USA; dilution both 1:200) were used. After incubation with the appropriate secondary antibody (Immunologic, Duiven, the Netherlands), slides were counterstained with Haematoxylin, dehydrated, cleared in xylene and mounted. All stainings were performed manually, with all individual samples processed in a single batch per staining.

Immunostained slides were all assessed and scored by a pathologist who was blinded to treatment allocation under identical light microscope conditions, including magnification (100x), gain, camera position, and background illumination.

### Histological evaluation

Inflammation was scored by a single blinded expert IBD-pathologist (SM) using the previously described Geboes-D’Haens histological inflammatory activity score [19]. In this score epithelial damage, architectural changes, infiltration of mononuclear cells and/or polymorphonuclear cells, crypt deformation, erosions, ulcers and granulomas are assessed. The score ranges from 0 (no inflammation) to 13 (maximum inflammation) points and was determined on the selected slides in the most involved region. In all patients this was located in the ileum.

For immunohistochemistry, staining intensity signals were determined on the most involved region of the selected slide using Image J analysis software (NIH, available free at rsbweb.nih.gov/ij). Results are given as the number of positive stained cells divided by the total surface area as indicated by haematoxylin staining. Positive staining was defined as moderate to strong unequivocal staining. Minimal expression was considered as background staining and not taken into account.

### RNA isolation, cDNA synthesis, and Quantitative Reverse-Transcription Polymerase Chain Reaction

For RNA isolation, 10 μm formalin-fixed, paraffin-embedded tissue sections were used. First the slides were stained for 30 seconds with Cresyl Violet in order to identify clearly the different layers of the intestine. For each patient, 10 full thickness sections were used and the mucosa and muscularis mucosa were separated from the submucosa and muscularis externa manually under the microscope using a 21G needle. RNA was isolated using the Qiagen RNA FFPE Kit (Qiagen, Valencia, CA). For the ileal biopsies, RNA was isolated using the Qiagen RNeasy Kit. RNA concentrations averaged around 200–300 ng/μL.

For cDNA synthesis, 10 μL RNA was transcribed using Revertaid (Fermentas, Vilnius, Lithuania). Quantitative RT-PCR was performed using SybrGreen (Qiagen) according to the manufacturer's protocol on a BioRad iCycler and primers from human qPrimer depot (http://primerdepot.nci.nih.gov/), or preoptimized primers from Sigma Aldrich. Expression relative to that of the control gene Acidic Ribosomal Protein 36B4 was calculated. Primer sequences can be found in S1 Table.

### Statistical analysis

Data were analysed using Graphpad Prism 5.0 (Graphpad Software Inc., La Jolla, CA). Descriptive data are given as mean and standard deviation (SD), or for non-parametric data as median and interquartile range (IQR). Kruskal Wallis test with post-hoc analysis or the Mann-Whitney-U test were performed to compare data with adjustment for multiple comparison by Bonferroni correction. For paired biopsy data a Wilcoxon signed rank test was performed. A p-value of < 0.05 was considered statistically significant.

## Results

### Patient characteristics

We identified 20 Crohn’s disease patients who were randomized to primary ileocecal resection and hence were naïve for IFX (IFX naïve group). Twenty other Crohn’s disease patients received IFX but did not respond -or were referred for resection from elsewhere because of non-response- after a median of 38 weeks, necessitating resections as well (IFX failure group). In both groups, one patient had prior exposure to Adalimumab (2 injections each). Baseline characteristics were comparable among the two groups (Table 1). Disease duration at time of surgery did not differ significantly with 84 months (IQR 30–301) in IFX naïve and 55 months (IQR 8–116) in IFX failure patients. CRP serum levels were comparable, indicating similar levels of systemic inflammation. Median time of previous thiopurines treatment did not differ significantly. The Montreal classification was also comparable, with the exception that significantly more perianal disease problems were seen in the patients who previously received IFX versus patients who were naïve to IFX (4 vs 0 patients, p = 0.04), Table 1.

**Table 1:** Baseline patient characteristics.

		IFX naïve	IFX failure
Number of patients		20	20
Age at operation (years, SD)		34,0 (13,6)	30,3 (12,5)
Female (%)		80%	80%
CRP pre-operative (mg/L, IQR)		14 (4–21)	9 (3–51)
Albumin (g/L, SD)		41 (+- 4)	38 (+- 6)
Duration IFX treatment (weeks, IQR)		-	38 (8–116)
Disease duration at time of surgery (months, IQR)		84 (30–301)	55 (35–140)
Time of previous thiopurines treatment (weeks, IQR)		22 (13–178)	6 (4–47)
Age at diagnosis (%)			
	- A1 (≤ 16 years old)	10%	15%
	- A2 (17–40 years old)	80%	80%
	- A2 (≥ 40 years old)	10%	5%
Crohn's disease behavior (%)			
	- B1 (non-stricturing, non-penetrating)	50%	40%
	- B2 (stricturing)	40%	45%
	- B3 (penetrating)	10%	15%
	- perianal disease	0%	20% (p = 0,04)
Crohn's disease location (%)			
	- L1 (ileum)	80%	85%
	- L2 (colon)	0%	0%
	- L3 (ileocolon)	15%	10%
	- L1 + L4 (ileum + upper GI)	5%	5%
Smokers (% yes)		35%	30%

SD: standard deviation, IQR: inter quartile range, CRP: C-reactive protein, IFX: Infliximab, GI: gastrointestinal tract

**Fig 1:**
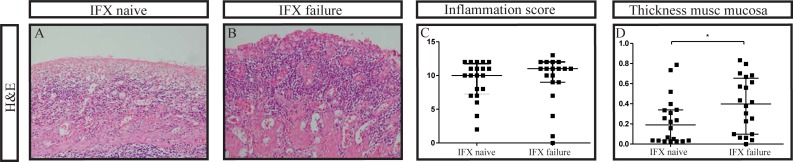
**Similar levels of inflammation in the intestine of patients naïve to Infliximab (A) compared to patients failed on Infliximab (B) (C)**. However patients failed on Infliximab have a significantly thicker muscularis mucosa as measured in millimeters (D). IFX: Infliximab. H&E: haematoxylin and eosin, original magnification in 1, 2 and 3: 100x, *p = <0.05. Median and interquartile range are shown in c and d.

Both patient groups had similar levels of intestinal inflammation as scored on H&E stained sections with the Geboes-D’Haens score with a median of 10 (IQR 7–12) points in the IFX naïve patient group and a median of 11 (IQR 9–12) in the IFX failure patient group (Fig 1). In combination with the serum CRP levels which did not differ significantly either, both patient groups were considered comparable for the severity of inflammation. Notably, the muscularis mucosa was significantly thicker in IFX failure patients as compared to IFX naïve patients, respectively 0.40 versus 0.19 millimeters, p = 0.04.

### Increased protein deposition of extracellular matrix component markers in IFX failure patients

**Fig 2:**
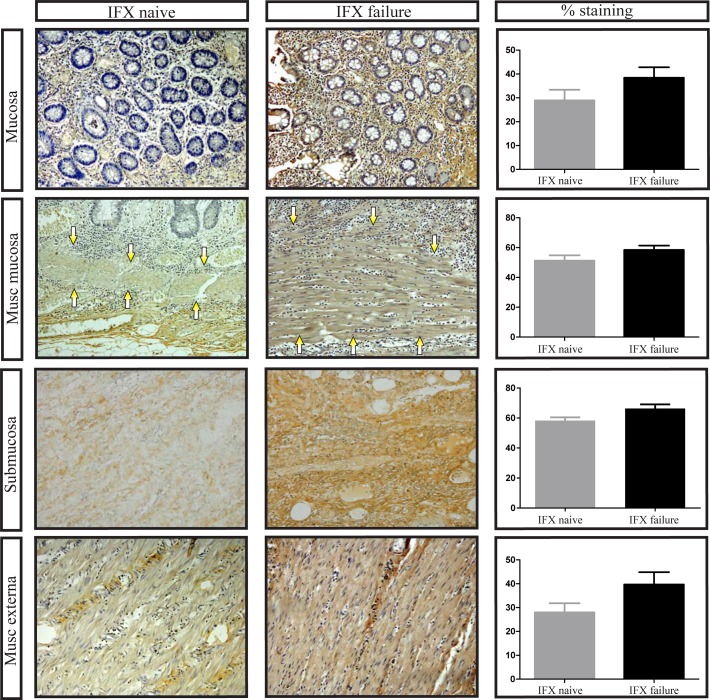
Increased collagen I expression in Crohn’s disease ileum failed on Infliximab treatment. Representative examples of collagen I staining in the mucosa, muscularis mucosa (arrows point to muscularis mucosa), submucosa and muscularis externa. Number of positive stained cells divided by the total surface area as indicated by haematoxylin staining. IFX: Infliximab, musc: muscularis, original magnification: 100x. Bars represent mean, error bars standard error of the mean.

**Fig 3:**
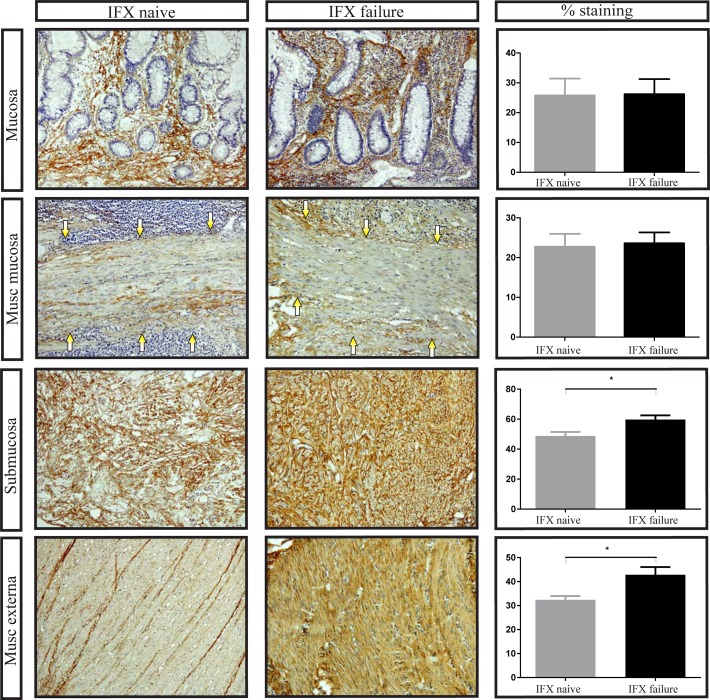
Increased collagen III expression in Crohn’s disease ileum failed on Infliximab treatment. Representative examples of collagen III staining in the mucosa, muscularis mucosa (arrows point to muscularis mucosa), submucosa and muscularis externa. Number of positive stained cells divided by the total surface area as indicated by haematoxylin staining. IFX: Infliximab, musc: muscularis, original magnification: 100x, * p = <0.05. Bars represent mean, error bars standard error of the mean.

We then investigated the deposition of the extracellular matrix markers fibronectin, collagen I and collagen III by immunohistochemistry. This was done in all layers of the intestinal wall, namely mucosa, muscularis mucosa, submucosa and muscularis externa. Crohn’s disease patients who had received prior treatment with IFX had more collagen I deposition compared to patients who were never treated with IFX, although this difference just did not reach statistical significance (Fig 2). Likewise, collagen III was more prominent in IFX failure patients than in IFX naïve patients, which was mainly seen in the deeper layers of the intestine (Fig 3).

**Fig 4:**
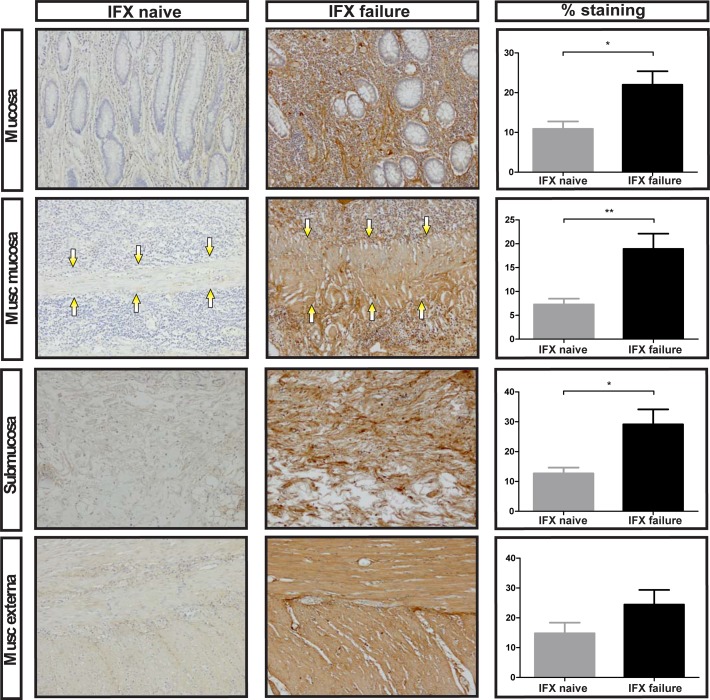
Increased fibronectin expression in Crohn’s disease ileum failed on Infliximab treatment. Representative examples of fibronectin staining in the mucosa, muscularis mucosa (arrows point to muscularis mucosa), submucosa and muscularis externa. Number of positive stained cells divided by the total surface area as indicated by haematoxylin staining. IFX: Infliximab, musc: muscularis, original magnification: 100x, * p = <0.05, ** p = < 0.01. Bars represent mean, error bars standard error of the mean.

Particularly there was more fibronectin deposition in patients who received previous IFX therapy compared to patients who never received IFX therapy (Fig 4). This difference reached statistical significance in the mucosa, muscularis mucosa, and submucosa (p = 0.022, p = 0.005 and p = 0.016, respectively).

### Elevated mucosal procollagen peptidase in IFX failure patients

**Fig 5:**
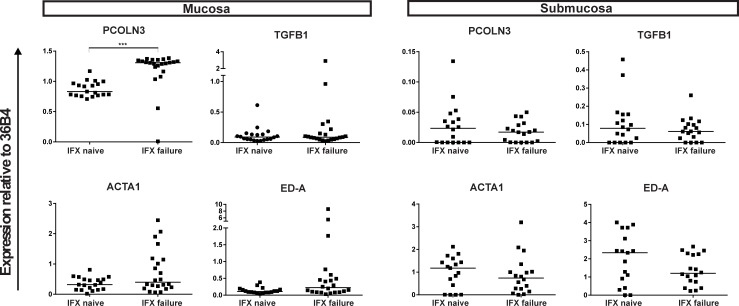
Relative expression of different genes of the mucosa and muscularis mucosa (= mucosa), and submucosa and muscularis externa (= submucosa) of Infliximab naïve patients compared to Infliximab failure patients. IFX: Infliximab, 36B4: Acidic Ribosomal Protein 36B4; ED-A: Extra Domain A; PCOLN3: procollagen peptidase; ACTA2: alpha smooth muscle actin; TGFB1: transforming growth factor beta 1. *** p = <0.001. All gene expressions are normalized against 36B4 as housekeeping gene.

**Fig 6:**
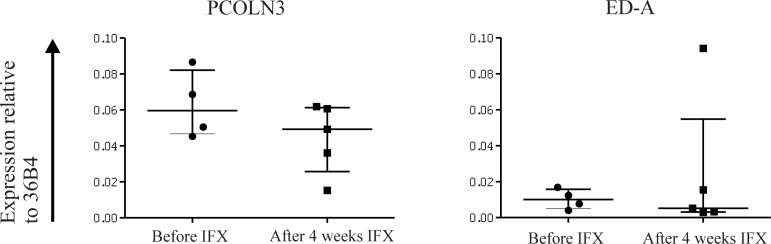
Similar expression of Extra domain A and procollagen peptidase in biopsies from Crohn’s disease patients right before Infliximab treatment and after 4 weeks of successful treatment. ED-A: Extra domain A; PCOLN3: procollagen peptidase; IFX: Infliximab; 36B4: Acidic Ribosomal Protein 36B4. All gene expressions are normalized against 36B4 as housekeeping gene.

We evaluated the levels of extracellular matrix modificators at transcriptomic level through isolation of mRNA from paraffin embedded tissues. Mucosa and muscularis mucosa (hereafter: mucosa) were separated from the submucosa and muscularis externa (hereafter: submucosa) meticulously as demonstrated by the significantly higher relative expression of the epithelial marker Keratin 18 as assessed by qPCR in the mucosa (S1 Fig). Fibrosis is characterized by increased expression of ECM molecules such as fibronectin and collagen. Collagen chains are synthesized by fibroblasts in precursor form as procollagens, translocated to the extracellular compartment and processed by procollagen peptidase to allow aggregation and deposition of collagen fibrils [20]. There was significantly more mRNA expression of procollagen peptidase (*PCOLN3*) in the mucosa of IFX failure patients compared to IFX naïve patients (p = <0.0001), indicating that more collagen accumulation had taken place in IFX failure patients (Fig 5). Transforming growth factor beta (TGFβ) is a dominant cytokine involved in fibrosis [21]. It induces expression of the Extra Domain A (ED-A) form of fibronectin, a variant that occurs through alternative splicing of the fibronectin transcript. Conversely, ED-A activates myofibroblasts which are characterized by expression of alpha smooth muscle actin (αSMA) and contribute to ECM deposition [22]. Transforming growth factor beta (*TGFB1*) mRNA expression was not significantly different in the mucosa and submucosa between the two patient groups. In the mucosa of IFX failure patients more alpha smooth muscle actin (*ACTA2*) mRNA expression was seen compared to the IFX naïve patients although this difference just did not reach statistical significance (p = 0.056). In the submucosa there was no difference in *ACTA2* expression seen between the two groups. Spliced variant of fibronectin, extra domain A (*ED-A*), showed a trend towards higher mRNA expression in the mucosa of IFX failure patients compared to IFX naïve patients (p = 0.08). In the submucosa no differences were observed in *ED-A* expression (Fig 5). As surgical material is not available for patients responding to the IFX therapy, expression levels before and after IFX treatment in responders was determined in a second cohort of 5 Crohn’s disease patients. Remarkably, there was no difference in mRNA expression of *ED-A* or *PCOLN3* in biopsies from Crohn’s disease patients who responded endoscopically to IFX treatment, compared to their own biopsies right before IFX treatment (Fig 6).

## Discussion

This study showed that Crohn’s disease patients treated with, but did not respond to, IFX display more fibronectin and collagen deposition in their intestine than patients naïve to IFX. One possible explanation for this may be that patients who did not respond sufficiently to IFX had already formed subclinical, microscopic fibrosis in the intestine due to chronic inflammation, and therefore did not respond to IFX therapy. Alternatively, the exposure to IFX itself may have altered the ECM. However, when we evaluated biopsies from 5 Crohn’s disease patients who did respond to IFX, expression of *ED-A* or *PCOLN3* was similar before and after exposure to IFX. Indeed in ileal biopsies from a cohort of Crohn’s disease patients in Leuven who were complete endoscopic responders or non-responders to IFX the same trend was observed [23]. In this cohort expression levels of *COL1A1*, *COL1A2*, *PCOLCE* (Procollagen C-Endopeptidase Enhancer) and *FN1* were higher in the non-responders compared to the responders before IFX treatment. Furthermore, expression of ECM associated genes decreased with response. Although it should be noted that the duration of anti-TNF exposure in this study was shorter than in our IFX failure group, exposure was sufficient to induce complete mucosal healing. Therefore, it appears unlikely that IFX itself leads to ECM formation.

Fibronectin plays an important physiological role during wound healing [24] and is responsible for the autocrine induction of myofibroblast migration [25]. On protein level, fibronectin was indeed increased in IFX failure patients. Additionally, fibronectin can occur in up to 20 different isoforms as a consequence of alternative splicing of the primary transcript, with different functions of the various forms [26]. Splicing variant ED-A is important for regulation of cell proliferation and differentiation of fibroblasts into myofibroblasts [22, 27]. One of the factors regulating the alternative splicing of fibronectin is tissue stiffness [28]. It can be hypothesized that due to increased levels of collagen and fibronectin, the tissue of IFX failure patients has increased tissue stiffness. Indeed increased levels of the ED-A splice variant were observed in IFX failure patients. This hypothesis is supported by the finding that the muscularis mucosa of those patients was significantly thicker than the mucosa of the patient group naïve to IFX.

Type I collagen is composed of several subunits, which are products of two genes, *COL1A1* and *COL1A2* and are secreted by fibroblasts into the extracellular space as procollagens. Procollagen peptidases cleave off the terminal peptides, enabling aggregation of collagen fibrils and deposition [20, 29, 30]. We found increased protein expression of both collagen I and III in the ECM of IFX failure patients, and on mRNA level procollagen peptidase was significantly more expressed in IFX failure patients. This argues for post-translational modifications of procollagen, leading to enhanced formation of collagen fibrils and accumulation of the collagen protein in the ECM.

The main limitation of this descriptive study is the fact that this is a retrospective analysis of prospectively collected data. Although the sections were processed via a protocolized approach where the sections were cut perpendicularly to the bowel wall, there was still some variation in the orientation and original localization of the section. However, this expected variation in the orientation was similar in the two groups and therefore it is unlikely that this has influenced the results. Moreover, due to the paucity of existing data regarding fibrotic markers under these circumstances, no power calculations were performed, resulting in a potential underpowering of the study. Nonetheless, these data provide a good indication for the differences found between the two groups, as well as the information required for power calculations in future studies. In addition, IFX exposure in this study was limited to non-responders. Ideally, one would compare responders to non-responders directly, but as responders obviously do not require surgery, resection specimens are not available for this group and a direct comparison is not feasible. Instead, we included patients randomized to surgery immediately and therefore were IFX naive. As allocation was performed randomly, the expected failure rate for this group is similar to that seen in the patients who were randomized to receive IFX (30% [17]). It should thus be noted that the IFX naive group cannot be considered as fully equal to an ‘IFX responder’ group. However, inclusion of potential non-responders among the IFX naïve group would only result in an underestimation of the differences between responders and non-responders and therefore does not invalidate our results.

Although this study did not allow determination of a causative relationship, our findings suggest that intestinal fibrosis is associated with insufficient response to IFX therapy in Crohn’s disease. Unfortunately, no biomarkers with predictive value for the development of intestinal fibrosis have been described to date. As our study evaluated surgical specimens, the direct translation of this data for prognostic use is limited. However, further evaluation of the use of (microscopic) fibrosis in biopsy material as a prognostic marker for response to anti-TNF seems warranted.

## Supporting information

S1 Fig Increased Keratin 18 expression in the mucosa compared to the submucosa of all patients together. KRT18: Keratin 18; 36B4: Acidic Ribosomal Protein 36B4. Gene expression is normalized against 36B4 as housekeeping gene. *** p = <0.001.
(TIF)

S1 Table Primer sequences. 36B4: Acidic Ribosomal Protein 36B4; ED-A: Extra Domain A; FN1: Fibronectin 1; PCOLN3: procollagen peptidase; ACTA2: alpha smooth muscle actin; TGFB1: transforming growth factor beta 1; KRT18: Keratin 18; Fw: forward; Rev: reverse.
(DOC)

## Abbreviations

Anti-TNFα: anti-tumor necrosis factor alpha
CD: Crohn's disease
ECM: extracellular matrix
IBD: Inflammatory bowel disease
IFX: Infliximab
α-SMA: alpha-smooth muscle actin
TGF-β: transforming growth factor-beta
