# Supplementary material for: Intestinal fibrosis is associated with lack of response to Infliximab therapy in Crohn's disease
Source: PLoS One. 2018 Jan 24;13(1):e0190999. doi: 10.1371/journal.pone.0190999 (PMC5783363; doi:10.1371/journal.pone.0190999)
Supplement: S1 Table — 36B4: Acidic Ribosomal Protein 36B4; ED-A: Extra Domain A; FN1: Fibronectin 1; PCOLN3: procollagen peptidase; ACTA2: alpha smooth muscle actin; TGFB1: transforming growth factor beta 1; KRT18: Keratin 18; Fw: forward; Rev: reverse. (DOC) [file pone.0190999.s002.doc]

**Supplementary table 1**

| Gene | Sequence |
| --- | --- |
| 36B4 | Fw TCATCAACGGGTACAAACGA  Rev GCCTTGACCTTTTCAGCAAG |
| ED-A | Fw GCTCAGAATCCAAGCGGAGA  Rev CCAGTCCTTTAGGGCGATCA |
| PCOLN3 | Fw GAGTCCTTCTCCGCCTTCTT  Rev GTGCTGCTCCTGTCATTGTG |
| ACTA2 | Fw CCAGAGCCATTGTCACACAC  Rev CAGCCAAGCACTGTCAGG |
| TGFB1 | Fw CTTCCAGCCGAGGTCCTT  Rev CCCTGGACACCAACTATTGC |
| KRT18 | Fw CGGGCATTGTCCACAGTATT  Rev GGGAGCACTTGGAGAAGAAG |

**Supplementary table 1**. Primer sequences. 36B4: Acidic Ribosomal Protein 36B4; ED-A: Extra Domain A; PCOLN3: procollagen peptidase; ACTA2: alpha smooth muscle actin; TGFB1: transforming growth factor beta 1; KRT18: Keratin 18; Fw: forward; Rev: reverse
